# Supplementary material for: Electro-Stimulation System with Artificial-Intelligence-Based Auricular-Triggered Algorithm to Support Facial Movements in Peripheral Facial Palsy: A Simulation Pilot Study
Source: Diagnostics (Basel). 2024 Sep 28;14(19):2158. doi: 10.3390/diagnostics14192158 (PMC11475571; doi:10.3390/diagnostics14192158)
Supplement: Supplementary file 1 [file diagnostics-14-02158-s001.zip › diagnostics-3195862-supplementary.pdf]

diagnostics

MDPI

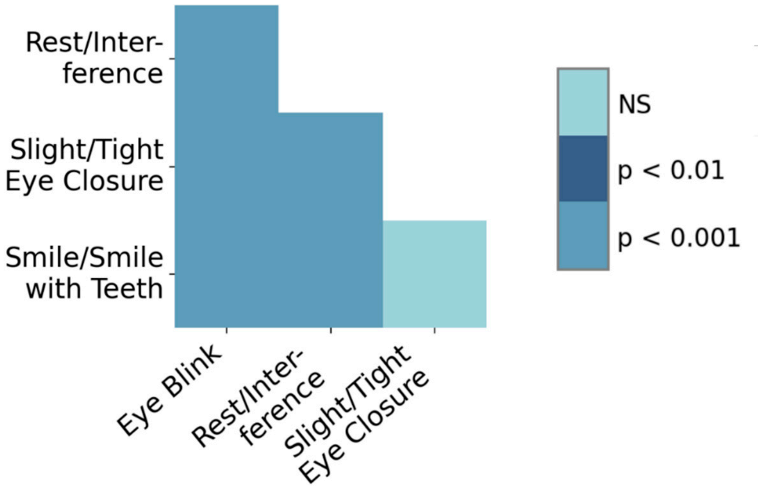

Supplement Figure S1. Results of the Conover post-hoc test for the test data. Visualizes, if there is a difference or not between the classes. NS stands for not significant.

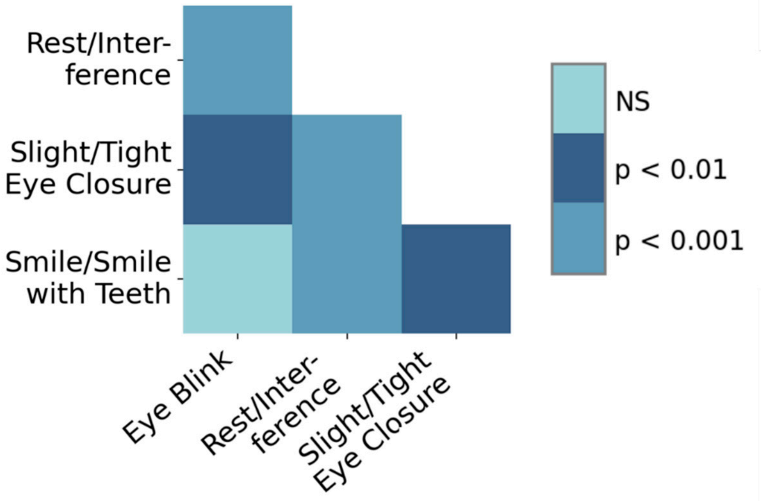

Supplement Figure S2. Results of the Conover post-hoc test for the everyday activities. Visualizes, if there is a difference or not between the classes. NS stands for not significant.
